# Living biobank-based cancer organoids: prospects and challenges in cancer research

**DOI:** 10.20892/j.issn.2095-3941.2021.0621

**Published:** 2022-07-21

**Authors:** Haixin Li, Hongkun Liu, Kexin Chen

**Affiliations:** 1 Cancer Biobank, Tianjin Medical University Cancer Institute & Hospital, National Clinical Research Center for Cancer, Key Laboratory of Molecular Cancer Epidemiology of Tianjin, Tianjin 300060, China; 2 Department of Epidemiology and Biostatistics, Tianjin Medical University Cancer Institute & Hospital, National Clinical Research Center for Cancer, Key Laboratory of Molecular Cancer Epidemiology of Tianjin, Tianjin 300060, China

**Keywords:** Cancer organoids, living biobanks, biobank, preclinical models

## Abstract

Biobanks bridge the gap between basic and translational research. Traditional cancer biobanks typically contain normal and tumor tissues, and matched blood. However, biospecimens in traditional biobanks are usually nonrenewable. In recent years, increased interest has focused on establishing living biobanks, including organoid biobanks, for the collection and storage of viable and functional tissues for long periods of time. The organoid model is based on a 3D *in vitro* cell culture system, is highly similar to primary tissues and organs *in vivo*, and can recapitulate the phenotypic and genetic characteristics of target organs. Publications on cancer organoids have recently increased, and many types of cancer organoids have been used for modeling cancer processes, as well as for drug discovery and screening. On the basis of the current research status, more exploration of cancer organoids through technical advancements is required to improve reproducibility and scalability. Moreover, given the natural characteristics of organoids, greater attention must be paid to ethical considerations. Here, we summarize recent advances in cancer organoid biobanking research, encompassing rectal, gastric, pancreatic, breast, and glioblastoma cancers. Living cancer biobanks that contain cancerous tissues and matched organoids with different genetic backgrounds, subtypes, and individualized characteristics will eventually contribute to the understanding of cancer and ultimately facilitate the development of innovative treatments.

## Introduction

**Figure 1:**
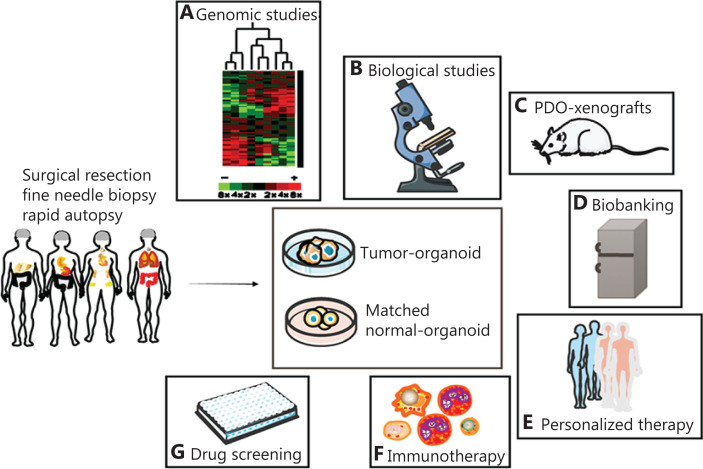
Application of patient-derived organoids. Organoids can be established from a patient-derived tumor and matched normal tissue. (A) Organoid cultures maintain genomic stability and the histology of original cancer tissues, and therefore can be exploited in genomic profiling analyses. (B) Organoids are useful tools for biological studies, because they recapitulate parent tissue histopathology characteristics. (C) Cancer organoids reproduce tumor tissue properties *in vivo* in murine xenografts and can thus be used in research on tumor metastasis. (D) Organoid biobanks are precious resources for oncology studies. (E) In personalized therapy, patient-specific organoids can help identify the best drug for each patient. (F) Coculture of tumor organoids with peripheral blood or T cells is feasible for expanding tumor-reactive T cells. (G) Cancer organoid lines could also be exploited to aid in the screening of anticancer drugs.

Organoids are miniaturized *in vitro* organ models developed from stem cells or tumor tissues extracted from patients in a specific 3D *in vitro* microenvironment. Organoids simulate the characteristics of real organs *in vivo* and can be stably expanded by 3D culture systems *in vitro*^1^. In 2019, the technology was designated as a “preclinical model of human disease”^2^. Organoids can be derived from various types of embryonic stem cells, induced pluripotent stem cells (iPSCs), somatic stem cells^3,4^, and patient-derived cancer cells^5^. In this review, we focus on patient-derived organoids (PDOs). Since Sato et al.^6^ first generated crypt-villus organoids from Lgr5(+) stem cells, organoid technology has become a promising tool in disease modeling. Many PDOs have been successfully constructed from breast cancer (BC), ovarian cancer (OC)^7–9^, lung cancer^10^, gastric cancer (GC)^11^, prostate cancer^12^, bladder cancer (BLC)^13^, liver cancer^14^, esophageal adenocarcinoma (EAC)^15^, pancreatic cancer (PC)^16^, neuroblastoma tumors, glioblastomas^17^, and colorectal cancer (CRC)^18,19^. Similarly, in the past 10 years, tumor organoids have become commonly used tools in oncology research (**Table 1** and **Figure 1**), including studies on cancer initiation and progression, preclinical models^20^, explorative and personalized therapies^21^, and drug screening^22^.

**Table 1:** Examples of application of tumor organoids

Organoid type	Translational research	Reference
Gastric cancer	Chemotherapeutic drug testing (5-FU, oxaliplatin, irinotecan, epirubicin, and docetaxel); targeted therapy testing (anti-HER2, imatinib, and palbociclib)	^100^
Metastatic colorectal cancer	Chemotherapeutic drug testing (irinotecan and 5-FU–irinotecan combination)	^101^
Colorectal cancer	High-content phenotypic drug screening (80 compounds either FDA approved or in clinical trials)	^97^
Esophageal adenocarcinoma	Chemotherapeutic drug testing (temozolomide and docetaxel); targeted therapy testing (CDK4/6 inhibitors and CDK2 inhibitors)	^102^
Prostate cancer	Ribosomal targeting drugs (CX-5461 and CX-6258)	^20^
Cholangiocarcinoma	Targeted drug testing (HSP90 inhibitor AUY922)	^103^
Colorectal cancer	Immunotherapy (generation of tumor-reactive T cells)	^104^
Pancreatic adenocarcinoma	Targeted epigenetic regulator testing (G9a inhibitor A366 and EZH2 inhibitor UNC1999)	^105^

	Basic research	

Colorectal cancer	Genetic cancer modeling	^106^
Gastric corpus tissues of mice	Inflammation-associated carcinogenesis	^107^
Neoplastic cerebral	Tumorigenic capability of gain- and loss-of-function mutations	^108^
Normal human colon	Mutational processes underlying cancer initiation and progression	^109^
Pancreatic ductal adenocarcinoma of mice	Tumor microenvironment	^110^
Progenitor organoids	Cancer modeling	^105^

## Construction of PDOs

Organoid construction involves many processes, and obtaining a sufficient number of tumor cells is a key factor for successful organoid construction. If the obtained tissue pieces contain muscle tissue or fat, they are removed to the greatest extent possible. Another key step is the digestion time, because overdigestion significantly decreases the growth efficiency of organoids. In addition, when a submerged method is used to culture organoids, plating should be performed as soon as possible after organoids are mixed with a basement membrane extract. The main components of the medium used for tumor organoid culture are divided into 2 categories: basal medium and various supplements.

### Main components of the culture medium

#### Basal medium

DMEM/F12 is used as the base medium in many studies, because it is suitable for clonal culture and is rich in nutritional factors.

#### Amino acids

Amino acids are the building blocks of proteins and one of the most basic conditions for organoid culture. Three amino acids are often added to the culture medium: L-glutamine, N-acetyl-L-cysteine, and nicotinamide. L-glutamine is an essential amino acid for cell growth. It can be used as an energy source for cultured cells, and it participates in protein synthesis and nucleic acid metabolism^23^. The GlutaMAX supplement provides an alternative to L-glutamine, because of its improved stability and promotion of cell health. N-acetylcysteine, a precursor of glutathione, is an effective antioxidant and free radical scavenger that protects cells against oxidative damage by regulating mitochondrial function^24^. Nicotinamide is a form of vitamin B3 that plays essential roles in cell physiology^25^ through facilitating NAD+ redox homeostasis and providing NAD+ as a substrate to a class of enzymes that catalyze nonredox reactions^26,27^; in addition, nicotinamide is an inhibitor of SIRT1. Nicotinamide-induced silencing of SIRT1 increases stem cell proliferation and differentiation^28,29^.

#### Cytokines

The Wnt proteins mainly affect stem/progenitor cells by regulating cell proliferation, differentiation, migration, and patterns during development^30^. Wnt-3A, a member of the Wnt family, is an activator of the Wnt/β-catenin pathway^31^ and is widely used in many organoid culture media. Wnt-activated organoids show tissue atypia, high proliferation and replication activity, prolonged culturability, and diminished apoptosis^32^. Wnt-3A is widely used for many types of cancer organoids and can be replaced by a Wnt-3A conditioned medium (**Table 2**).

**Table 2:** Summary of culture protocols for various cancer organoid types

Components		CRC		NSCLC	NT	LC		PC	BC	GC		BLC	OC	CC	PTC		EAC	HNSCC
Base medium	Advanced DMEM/F12	√	√	√	DMEM-GlutaMAX	√	√	√	√	√	√	√	√	√	√	DMEM/F12	√	√
	HEPES	1%	10 mM			10 mM	10 mM	1×	10 mM	10 mM			12 mM	10 mM		10 mM	1×	
	GlutaMAX	1%	2 mM			1%	1%	1×	1×	2 mM			1%	1×			1×	
	L-Glutamine										2 mM							
	Penicillin/streptomycin	1%	√		100 μg/mL	1%	1%	1×	100 μg/mL	√	100 units/mL						1×	
	N-acetyl-L-cysteine	10 mM	1 mM	1.25 mM		1.25 mM	1.25 mM	1 mM	1.25 mM	1 mM		1.25 mM		1.25 mM	1.25 mM		1 mM	1.25 mM
	Nicotinamide	10 mM		10 mM		10 mM	10 mM	10 mM	5 mM		4 mM	10 mM	1 mM	2.5 mM	10 mM	100 μM	10 mM	10 mM
	B27	1×	1×	1×	√	2%	2%	1×	1×	1×	1×	2%	2%	1×	1×	0.5×	1×	1×
	Primocin	100 μg/mL						1 mg/mL	50 mg/mL					100 μg/mL			1 mg/mL	
Cytokine	R-Spondin									1 mg/mL	500 ng/mL				500 ng/mL			4%
	R-Spondin conditioned medium	20%	10%	10%			10%	10%	10%				25%	10%			20%	
	Noggin						25 ng/mL		100 ng/mL	100 ng/mL	100 ng/mL		100 ng/mL		100 ng/mL	25 ng/mL	0.1 μg/mL	4%
	Noggin conditioned medium	10%		10%				10%						1%				
	rmNoggin		100 ng/mL															
	Wnt-3A										100 ng/mL							
	Wnt surrogate													0.5 nM				
	Wnt-3A conditioned medium		50%				30%	50%		25%			25%				50%	
	rhEGF	50 ng/mL			20 ng/mL	50 ng/mL	50 ng/mL	50 ng/mL	5 ng/mL				10 ng/ mL	50 ng/μL				50 ng/mL
	EGF														50 ng/mL	20 ng/mL	50 ng/mL	
	rmEGF		50 ng/mL							50 ng/mL	50 ng/mL							
	rhHGF					25 ng/mL	25 ng/mL											
	rhFGF10			100 ng/mL		100 ng/mL	100 ng/mL	100 ng/mL	20 ng/mL	50 ng/mL	10 ng/mL	100 ng/mL	100 ng/mL		10 ng/mL		100 ng/mL	10 ng/mL
	FGF										10 ng/mL							
	rhFGF7			25 ng/mL					5 ng/mL			25 ng/mL		25 ng/mL	5 ng/mL			
	FGF2				40 ng/mL							12.5 ng/mL				20 ng/mL		5 ng/mL
	PDGF-AA				10 ng/mL													
	PDGF-BB				10 ng/mL													
Small molecule compound	VEGF-121															10 ng/mL		
	IGF-1				200 ng/mL													
	Gastrin	10 nM	10 nM			10 nM	10 nM	10 nM		10 nM	10 nM							
	PGE2	10 nM									1 μM							1 μM
	Neuregulin 1								5 nM									
	SB202190	3 μM	10 μM	1 μM					500 nM		5 μM				10 μM		10 μM	
	SB431542												0.5 μM					
	Y-27632			5 μM		10 μM	10 μM		5 mM		10 μM	10 μM	9 μM	10 mM		10 μM		10 μM
	A83-01	500 nM	500 nM	500 nM		5 μM	5 μM	0.5 μM	500 nM	500 nM	0.5 μM	5 μM		500 nM	500 nM	5 μM	0.5 μM	500 nM
	CHIR 99021													0.3 μM				3 μM
Other supplements	N2				√	1%	1%				1×		1%					
	Forskolin					10 μM	10 μM							10 mM				1 μM
	β-Estradiol													100 nM				
	DEX					3 nM												
References		^104,111,112^	^113^	^104^	^114^	^14^		^115,116^	^59^	^82^	^57^	^91^	^9^	^117^	^118^	^119^	^15^	^120^

NSCLC, non-small cell lung carcinoma; NT, neuroblastoma tumor; EGF, epidermal growth factor; FGF, fibroblast growth factor; IGF, insulin-like growth factor; PDGF, platelet-derived growth factor; LC, liver cancer; PTC, papillary thyroid cancer; VEGF, vascular endothelial growth factor; HNSCC, head and neck squamous cell carcinoma; CC, cervical cancer.

Lgr4, Lgr5, and Lgr6 function as receptors for ligands of the R-spondin family, which are essential Wnt signal enhancers in multiple adult stem cell compartments^33^. Expansion of tissue-specific adult stem cells from a variety of endoderm-derived organs, including the stomach, small intestine, and colon, requires the activation of Lgr5 by its ligand R-spondin^34^. As an activator of the Wnt signaling pathway, R-spondin1 plays a supporting role in the development and growth of breast organoids, small intestine organoids, liver organoids^14^,^35^, and pancreas organoids^36^. Some reports have also indicated that R-spondin1 can be replaced by an R-spondin1 conditioned medium (**Table 2**).

Noggin is also a stem cell niche factor, and it is beneficial for long-term culture of organoids^34,37^.

Epidermal growth factor (EGF) plays an important role in regulating cell growth, proliferation, and differentiation. Evidence indicates that EGF promotes the proliferation and differentiation of fallopian tube organoids^38^. In addition, EGF is widely used for liver organoids^35^, BC organoids^39^, and intestinal organoids^40^.

The fibroblast growth factor (FGF) family is a family of growth factors with a wide range of physiological functions, including promoting cell mitosis and survival, and the growth of mesoderm and neuroectoderm cells. FGF2, FGF7, and FGF10 are widely used in organoid culture. Studies have shown that the combination of insulin-like growth factor 1 (IGF-1) and FGF2 enhances the clonogenic capacity of human intestinal stem cells^41^.

Hepatocyte growth factor (HGF) promotes hepatocyte proliferation and has been widely used in liver organoid culture^42–44^.

#### Small molecule compounds

CHIR99021, an inhibitor of glycogen synthase kinase 3, promotes the proliferation of iPSCs^45^ and modulates cerebral organoid development through dose-dependent regulation of apoptosis, proliferation, differentiation, and migration^46^.

Y-27632 inhibits the apoptosis of embryonic stem cells by inhibiting Rho-associated, coiled-coil containing protein kinase, and it promotes the self-renewal and proliferation of stem cells^47^. Studies have suggested that Y-27632 decreases caspase-dependent cell apoptosis^48^ and promotes the formation of organoids^49–51^.

A83-01, an effective inhibitor of TGF-β type I receptor (ALK5-TD), promotes cell proliferation^52^ and colonosphere formation^53^. Y-27632, A83-01, and CHIR99021 are often used together to improve cell survival and cell proliferation^51,54^.

SB431542 is an inhibitor of the TGF-β/activin signaling pathway. Evidence indicates that SB431542 promotes the differentiation of iPSCs and embryonic stem cells into mesenchymal-like cells^55^.

SB202190 is an inhibitor of mitogen-activated protein kinase p38; it promotes intestinal organoid formation and enterocyte differentiation^56^, and has been used for gastrointestinal cancer organoids^57^, BC organoids^58^, and PC organoids.

The addition of specific small molecule compounds is often required for different types of organoids. For example, neuregulin 1 is required for BC organoids, because it is a ligand of human EGF receptor tyrosine kinases-3 and -4, and it promotes the generation and long-term expansion of BC organoids^59^. Prostaglandin E2 (PGE2) must be added to the culture of liver organoids^43,44^ and prostate organoids^60–62^, and gastrin is required for gastric organoid culture. β-Estradiol has a wide range of applications in OC organoids and cervical cancer organoids. Dexamethasone (DEX), a synthetic corticosteroid, is a clinical immunosuppressant. Evidence has indicated that DEX improves the survival of retinal organoids^63^.

### Other supplements

As a serum-free medium additive, B27 is suitable for stem cell culture and most nerve cell cultures. As summarized in **Table 2**, almost every type of organoid culture medium contains B27. The function of the N2 supplement is similar to that of B27. Forskolin activates adenosine cyclase, thus increasing the levels of cAMP, and is often used for liver organoids.

## Why PDO living biobanks are required

Despite many technological and research advances, cancer remains a major global threat to public health. In addition, the cancer burden is expected to disproportionately affect less developed countries in the future. In particular, the incidence rate of cancer in moderately developed and underdeveloped countries is expected to increase by an estimated 100% and 81%, respectively. Beyond timely prevention and early diagnosis, more innovative targeted therapies for cancer prevention and treatment are urgently required. However, drug research and development are not straightforward processes; many regulatory bottlenecks limit the rapid transformation of innovative science into effective therapies. One such issue is that cancer models fail to replicate the tissue complexity and genetic heterogeneity observed in tumors^64^.

At the genomic level, two-dimensional monolayer cell cultures are predominantly used to study tumor growth and drug sensitivity *in vitro*; however, this model lacks several tumor characteristics, including hypoxia^65^, cell–matrix interactions, and cell–cell interactions^66^.

Because of the high genetic homology between mice and humans^67^, the mouse genome can be manipulated to investigate and determine gene function during cancer development^68,69^. As a tool for oncology research, genetically engineered mouse models (GEMMs) offer several advantages. Tumors often naturally arise *de novo* in the normal immune system^70^; therefore, GEMMs can mimic histopathological and molecular tumor characteristics. However, the cancer stroma is completely distinct from the normal stroma, and is key for supporting the generation and growth of cancer cells^71,72^. Although the construction of GEMMs in embryonic stem cells is expensive and time consuming, the spontaneous tumorigenesis cycle is difficult to synchronize, owing to interindividual differences among animals. Similarly, GEMMs usually contain 1 or 2 gene mutations, whereas human tumors may have multiple mutations in multiple genes^73^.

As a traditional preclinical model, patient-derived xenografts (PDXs) have been widely used to determine drug sensitivity^74,75^ for individual patients. Because tumors are directly obtained from patients with cancer, they elicit tumor characteristics, including histologic, genetic, and phenotypic features. Despite their advantages, PDXs have several limitations, as follow. (1) Their transplantation rate varies among cancers^76^, and higher transplantation rates have been observed for gastrointestinal tumors, whereas lower rates have been observed for BC tumors^77^. Given that cancers are naturally heterogeneous, implanted microscopic tumor masses do not faithfully represent primary tumor characteristics^78^. (2) PDX construction requires 1–3 months or longer^79^. (3) Immunodeficient mice are expensive to house in strict, dedicated, clean environments until the necessary tumor size has developed^80^. (4) PDX is unsuitable for studying tumor initiation.

Given the limitations of such traditional preclinical models, organoid cultures have emerged as a viable *in vitro* alternative to these models and have many advantages (**Table 3**). Thus, the establishment of a PDO living cancer biobank can accelerate understanding of cancer. First, unlike establishing cell lines or PDXs, which can require several months, organoids can be established and tested within several days after collection. This rapid timeframe ensures that the organoids are as histologically and genomically similar to the primary tumor as possible, thus yielding more accurate results. Second, PDO models are an important step in improving precision oncology. Researchers must develop a sufficient number of high-quality organoid models to accurately represent the most common cancer subtypes. Third, cancer organoids can be grown for as long as 6 months or cryopreserved and kept at −80 °C in live organoid biobanks for future research purposes, thus filling a gap in preclinical cancer models. Fourth, PDO living biobanks can integrate clinical, genomic, and functional data from multiple sources, including patient-derived tumor organoids, to identify potential therapeutic targets. Fifth, cancer organoid models can help researchers overcome several challenges in research on rare cancers. The rarest cancers may lack established primary treatments, and years may be required to enroll a sufficient number of patients to complete a clinical trial. Organoid models can accelerate this research by serving as a platform for high-throughput drug screening.

**Table 3:** Compared with the traditional *in vitro* model, organoids have more advantages and greater potential, and play an essential role in tumor basic and clinical research

Features	Organoids	Cell lines	PDX
Biobanking	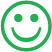	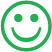	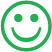
Multidimensionality	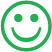	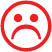	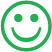
Retention of heterogeneity and mutations	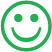	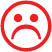	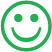
Abundant gene expression subtypes	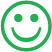	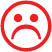	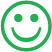
Matched normal controls	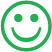	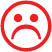	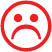
Multiple cell types	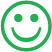	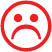	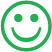
High-throughput drug analyses	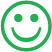	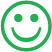	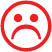
Easy genetic modification	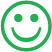	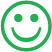	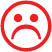
Mimicking *in vivo* cell environments	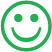	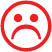	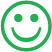
High concordance of somatic mutations with those in the primary tumor	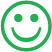	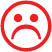	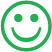
Retention of the heterogeneity of the primary tumor	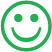	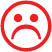	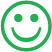
Stable genetic and phenotypic features	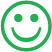	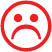	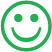
Ability of tumor cells to interact with the stroma	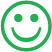	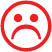	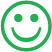
Low cost	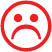	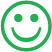	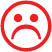
High proliferation rates	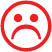	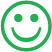	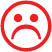

## PDO living cancer biobanks and their applications

PDO living cancer biobanks, in contrast to traditional cancer biobanks, can store viable and functional biospecimens. Theoretically, PDOs can be collected from any tumor developmental stage; however, only a small fraction of tumor tissues can be cultured and expanded *in vitro*. PDO biobanks serve as a practical platform for basic medicine and clinical science oncology research. Joshi et al.^81^ have analyzed the DNA methylation signatures of 5 tumor organoids from the American Type Culture Collection and concluded that the organoid DNA methylation profiles not only maintain the epigenetic signature characteristics of the original primary cancer but also have more corresponding primary tumors than established two-dimensional-culture cell lines. A Dutch Nonprofit Technology Group Hub (https://huborganoids.nl/) has established an organoid biobank similar to the American Type Culture Collection, with more than 800 different organs, including known genetic information and other characteristics. In addition, many laboratories are trying to establish PDO living cancer biobanks (**Table 4**).

**Table 4:** Characteristics of living biobank-based cancer organoids in recent years

Type	Primary/metastatic	Methods	Generation efficiency	Clinical stage (number)	Treatment condition	Pathological diagnosis	Reference
Pancreatic cancer	Primary and metastatic	Surgical resection, fine needle biopsy, rapid autopsy, and video-assisted thoracoscopic surgical resection	75% (72% for fine needle biopsy, 78% for tumor resection)	I (1); II (34); III (7); IV (24)	12 generated from 5 pretreated patients; 57 generated from 55 patients who did not receive treatment	Not described	^88^
Breast cancer	Primary and metastatic	Not described	>80%	I (13); II (45); III (29)	72 patients did not receive chemotherapy; 9 received chemotherapy	Malignant neoplasm	^59^
Multitype cancer	Primary and metastatic	Surgical resection	73%	I–III (exact number is unclear)	Not described	Colon (metastatic adenocarcinoma, invasive low-grade adenocarcinoma, and moderately differentiated adenocarcinoma); kidney (renal cell carcinoma and clear cell carcinoma); lung (moderately differentiated invasive squamous cell carcinoma, invasive squamous cell carcinoma, invasive poorly differentiated squamous cell carcinoma, well-differentiated adenocarcinoma, and invasive metastatic adenocarcinoma); pancreas (moderately differentiated adenocarcinoma, invasive moderately differentiated ductal adenocarcinoma, and moderate-to-poorly differentiated metastatic adenocarcinoma)	^94^
Colorectal and gastroesophageal cancer	Metastatic	Ultrasound-guided, computed tomography-guided, and endoscopic biopsy	70%	II (1); III (11); IV (17)	All patients received	Not described	^57^
Colorectal cancer	Primary and metastatic	Endoscopic biopsy and surgical resection	100%	I (5); II (10); III (3); IV (10); not available (3)	Only 3 received	Adenocarcinoma, neuroendocrine neoplasms, and premalignant lesions	^83^
Rectal cancer	Not described	Endoscopic biopsy	85.7%	Not described	Did not receive	Adenocarcinoma, mucinous adenocarcinoma, and mucinous adenocarcinoma with signet ring cell carcinoma	^87^
	Primary and metastatic	Endoscopic biopsy	77%	Not described	22 derived from treatment-naive patients; 43 derived from patients undergoing first- or second-line therapy	Not described	^86^
Childhood kidney cancer	Primary and metastatic	Surgical resection and biopsy	75% for Wilms tumors, 100% for malignant rhabdoid tumors, 75% for renal cell carcinomas	Not described	Most received	Wilms tumor, malignant rhabdoid tumor, renal cell carcinoma, congenital mesoblastic nephroma, metanephric adenoma, and the rest nephrogenic	^85^
Urothelial cancer	Not described	Surgical resection and biopsy	Approximately 50%	Low grade and high grade	Some received chemotherapy or radiotherapy	Squamous cell carcinoma, papillary urothelial cell carcinoma, expansive necrotic invasive urothelial cell carcinoma, poorly differentiated transitional cell carcinoma with glandular squamous differentiation, and papillary nonmuscle invasive urothelial cell carcinoma	^91^

### Mechanisms of tumorigenesis and development

The PDO living cancer biobank is a valuable platform for studying the molecular mechanism of gastric carcinogenesis. Nanki et al.^82^ have established a library of GC organoids from 37 patients, which contains various lesions (primary tumors, metastases, and carcinomatous ascites) and histological types, including poorly differentiated adenocarcinoma, signet ring cell carcinoma, and hepatoid adenocarcinoma, as well as 2 matched normal organoids, 7 nontumor organoids, and 9 normal-like organoids. Through modification of the culture protocol, their success rate reached 74.6%. The establishment rate of GC organoids did not differ among histopathological subtypes. Combining a PDO living biobank with genetic engineering has provided insights into histopathological transformation during human gastric tumorigenesis. CDH1 in diffuse-type GC has been shown to be repeatedly mutated regardless of the molecular subtype, and both RHOA mutations in solid GC organoids are heterozygous and found at recurrently mutated positions. Normal gastric organoids subjected to CRISPR–Cas9-induced knockout (KO) of the CDH1 and/or RHOA genes exhibits different morphologies. Whereas RHOA^KO^ organoids maintain a normal cystic morphology, CDH1^KO^ organoids show a solid structure with vigorous migratory activity. To further study the genotype-phenotype correlation in GC, the authors focused on the relationship between niche factor dependence and gene mutation. For example, GC organoids with a single ERBB3 or PTEN mutation still depended on EGF and FGF, whereas GC organoids with ERBB2 or ERBB3 amplification were invariably EGF/FGF10 independent.

### High-throughput drug screening

Vlachogiannis et al.^57^ have generated a living PDO biobank derived from patients with metastatic, heavily pretreated CRC and gastroesophageal cancer recruited in a phase I/II clinical trial, and have achieved a 70% success rate. The success rate was not correlated with necrosis but was strongly correlated with tumor cellularity in the parental biopsy. The authors tested 55 drugs, including conventional chemotherapy and targeted drugs (antibodies and small molecule inhibitors) currently in phase I–III clinical trials, by using 3D high-throughput drug screening, and revealed a 100% sensitivity, 93% specificity, 88% positive predictive value, and 100% negative predictive value for forecasting the responses of 21 paired patients. Because the statuses of participants in nonrandomized controlled clinical trials and patients differ, 21 paired PDOs and primary tumors were compared. To evaluate the ability of PDOs to recapitulate responses to regorafenib, the authors generated PDO xenograft models. Whereas regorafenib did not affect PDOs, it inhibited angiogenesis in PDO xenografts, thereby suggesting that vessel cooperativity is a mechanism underlying primary resistance to regorafenib. Thus, PDOs must be combined *ex vivo* and *in vivo*. PDOs are not only valuable for drug prediction but also reflect the ability of cancers to evolve after treatment. Xenografts were generated from the same liver metastatic tissues before (BL) and after treatment (PD) in patients with mCRC, and the microvasculature region in BL PDO xenografts decreased by approximately 60% in response to regorafenib, whereas that in the PD PDO xenografts did not decrease.

Sachs et al.^59^ have conducted high-throughput drug sensitivity screens by using a BC organoid biobank. Most BC organoids with high expression of HER2 were sensitive to drugs targeting the HER2 signaling pathway, whereas HER2-negative organs were insensitive. BC organoids with high BRCA1/2 signatures were sensitive to PARP inhibitors, whereas BC organoids with low BRCA1/2 signatures were not.

### Correlation between disease progression and niche factors

Fujii et al.^83^ have generated a comprehensive organoid library of various histological cancer subtypes and clinical stages from patients with CRC. The authors have observed that organoid cultures required not only niche factors (combinations of EGF, Noggin, A83-01, and SB202190) but also Wnt activators, appropriate oxygen concentrations, and p38 inhibitors. With this modified protocol, the model efficiency was 100%, except in cases of bacterial contamination and samples with massive necrosis. The library consisted of 55 organoids derived from 52 common subtypes and rare histological tumor subtypes, and 41 matched normal organoids. The niche factor requirements varied substantially among organoids, and the differences in niche factor requirements were determined predominantly by genetic mutations, and contributed to local tumorigenicity. For example, adenoma organoids required EGF instead of Wnt3A/R-spondin 1, whereas the dependence of patients with microsatellite-stable CRC on Noggin, A83-01, and normoxic culture conditions was reduced. Benign tumor and CRC organoid lines were xenotransplanted into the kidney subcapsules of NOG mice, and the resulting tumorigenesis profiles were completely different. Successful tumor formation was observed in all CRC cases, whereas benign tumor organoids exhibited no or substantially minimal engraftment. Moreover, the size of the engrafted subrenal CRC tumors correlated with niche factor requirements.

A pancreatic tumor and normal organoid library derived from 39 patients with pancreatic ductal adenocarcinoma (PDAC) has been established by Seino et al.^84^. The authors mainly focused on stem cell niches during tumorigenesis, and identified several functional PDAC organoid subtypes (normal-like organoids, Wnt-nonproducing organoids, Wnt-producing organoids, and Wnt- and R-spondin-independent organoids) with distinct Wnt niche requirements. Among these organoids, normal-like and Wnt-nonproducing organoids required exogenous Wnt and R-spondin ligands. Wnt-producing organoids were independent of exogenous Wnt ligands but relied on R-spondin, and Wnt and R-spondin-independent organoids had no requirement for Wnt signal activation. Further experiments showed that niche independency was mainly acquired by driver gene mutations, whereas Wnt niche independency was regulated predominantly by epigenetic mechanisms. For example, CRISPR–Cas9 genome-edited organoids carrying driver gene mutations in KRAS (K), TP53 (T), CDKN2A (C), and SMAD4 (S) have different proliferation profiles in the absence of Wnt3A. Kidney cancer (KC) and KT organoids ceased to proliferate and became extinct within 1–3 weeks without Wnt3A, whereas KCT and KCTS organoids stopped proliferating but survived and grew without exogenous Wnt3A after multiple passages. GATA6 expression was highest in Wnt-nonproducing organoids and lowest in Wnt- and R-spondin-independent organoids. In contrast, Wnt-nonproducing PDAC organoids exhibited higher methylation levels of WNT10A than the other subtypes.

Calandrini et al.^85^ have conducted high-throughput drug screens (150 compound libraries) by using a KC organoid biobank. A variety of MEK and HDAC inhibitors were found among the 25 most effective compounds. Wilms tumor organoids are more sensitiveto panobinostat (a pan-HDAC inhibitor), whereas normal kidney organoids did not, thus suggesting that the therapy appears to be less toxic.

### Prediction of patient responses to treatment

The establishment of tumor and normal organoids from biopsied specimens is highly important, because it allows for model establishment at the time of diagnosis, throughout the entire treatment period, and at recurrence. Patients’ specific and clinically related reactions to chemotherapy, radiotherapy, and targeted therapies can be summarized, thus aiding in the selection of better treatment methods. Ganesh et al.^86^ have constructed a library of 65 RC organoids from 41 patients with primary, metastatic, or recurrent disease, achieving a 77% success rate, and a library of 51 normal rectal organoids from normal adjacent tissue. Of the 65 RC organoids, 43 were derived from patients undergoing first- or second-line therapy; therefore, clinical treatment did not influence the success rate of cancer organoid establishment. Of note, 49 of the RC tumoroids were established from biopsied tissue. These findings indicate that biopsies can be performed as a standard of care in pretreatment and posttreatment settings to generate tumoroid models to assess patient-specific responses and resistance mediators. Importantly, RC organoids recapitulated patient-specific and clinically relevant responses to chemotherapy, radiation, and targeted therapy. Additionally, these mini-organs could be grafted into the rectum in mice to evaluate invasion capabilities and eventual metastasis. In another RC organoid biobank of 80 tumor organoids^87^, tumor organoids were shown to accurately predict the efficacy of neoadjuvant (NACR) radiotherapy and chemotherapy of locally advanced RC. For example, when the organoid radiosensitivity data were matched with patient clinical outcomes, 16 patients with organoids sensitive to irradiation were found to respond well to NACR treatment; moreover, among the 64 patients whose organoids were resistant to irradiation, 42 had a poor response to NACR, and 22 had a good response. For 5-fluorouracil (5-Fu), 27 patients whose organoids were sensitive to 5-Fu responded well; of the 53 patients with PDOs resistant to 5-Fu, 38 had a poor response, and 15 had a good response. In addition, 32 PDOs were sensitive to CPT-11, and these patients had a good response; of the other 34 patients with organoid resistance to CPT-11, 27 had a poor response, and 7 had a good response. Moreover, the author compared the PDO responses to the clinical outcomes of the 80 patients who received NACR. The combined PDO data highly correlated with the patients’ clinical outcomes, with an AUC of 88.20%, an accuracy of 84.43%, a sensitivity of 78.01%, and a specificity of 91.97%.

Tiriac et al.^88^ have obtained 159 PDAC samples from primary tumors and metastases of 138 patients for PDO generation, achieving a success rate of 75%. In this experiment, organoids were treated with 5 chemotherapeutic drugs (gemcitabine, paclitaxel, irinotecan, 5-Fu, and oxaliplatin) to investigate the relationship between PDO pharmacotyping and patient treatment responses. PDOs generated from different patients showed heterogeneity in the chemotherapy response. Among these cases, the longitudinal PDO of one patient reflected the clinical stage. The first organoid (hM1A) was generated from resection of a lung metastasis, and this patient responded well to 5-Fu, leucovorin, irinotecan, oxaliplatin, and gemcitabine/nab-paclitaxel regimens. Correspondingly, the PDO was sensitive to gemcitabine, paclitaxel, 5-Fu, and oxaliplatin, and exhibited an intermediate irinotecan response. Approximately 2 years later, the patient’s condition deteriorated, and histological analysis revealed neuroendocrine/small cell-like characteristics; organoids (hM1E) were generated at that time. The final organoid (hM1F) was established shortly after the patient succumbed to the disease. The hM1E and hM1F PDOs were resistant to gemcitabine, paclitaxel, and irinotecan, whereas hM1F gained additional resistance to oxaliplatin and switched to a more basal-like subtype. In the chemosensitivity assessment of PDAC-confirmed PDO cultures, 22 organoid lines lacked sensitivity to all 5 chemotherapeutic agents; therefore, the authors used alternative treatment strategies for the 22 PDO cultures, with a panel of targeted agents (*n* = 21). Finally, they observed that half of the 22 organoids were extremely sensitive to targeted drugs.

To mimic the postsurgical standard of care treatment, Jacob et al.^89^ have exposed 8 glioblastoma organoid (GBO) samples from 7 patients to a single dose of radiation at 10 Gy in combination with temozolomide treatment for 1 week. GBOs from 3 of 7 patients exhibited decreased percentages of KI67^+90^ cells after temozolomide and radiation treatment, and one patient with decreased GBO KI67^+^ cell numbers exhibited a radiographic decrease in tumor volume after 1 month of treatment for recurrence. Moreover, the survival times of 3 patients whose GBO KI67^+^ cell numbers did not significantly change after treatment were below the median.

For bladder cancer, 3 organoid lines were selected from the Human Bladder Cancer Biobank^91^ and exposed to 6 first-line chemotherapeutic agents. Differences were found among the lines. For example, the organoid line HBL7T2 was relatively resistant to epirubicin and doxorubicin. In contrast, the organoid line HBL12N was more sensitive to gemcitabine and vincristine.

### Possibilities for immunotherapy

In recent years, immunotherapy research has progressed, but, because of the complexity of the tumor microenvironment, the efficacy of *in vitro* models of immunotherapy drugs has not been matched in clinical settings^92,93^. PDO technology provides a technical breakthrough in this regard and may make immunotherapy possible in the near future.

Using an air-liquid interface method, Neal et al.^94^ have established more than 100 organoids from 100 individual patient tumors, representing 14 distinct tissue sites and 28 unique disease subtypes, with a 73% success rate. The authors selected 15 of the most rapidly growing organoids for cryopreservation and observed that 80% could be cryo-recovered and serially repropagated every few weeks. Importantly, the organoids included preserved fibroblast stroma and diverse immune elements comprising macrophages, CD8+ T cells, CD4+ T cells, B cells, natural killer cells, and natural killer T cells. Furthermore, the PDOs faithfully recapitulated the TCR repertoire of the original tumors, and tumor-infiltrating lymphocytes functionally recapitulated PD-1-dependent immune checkpoint mechanisms. Together, these data suggest that organoids represent the advent of precision cancer therapies.

Personalized CAR-T immunotherapy has been verified in GBOs^89^. In that article, the authors investigated the coculture of CAR-T cells with 6 GBO samples, including GBOs with high and low percentages of EGFRvIII+^95^ cells, and 2 pairs of GBOs from different tumor regions, in which one subregion contained a high percentage of EGFRvIII+ cells and the other subregion did not. The results showed marked expansion of CAR-T cells only after incubation with EGFRvIII+ GBOs. The increased CAR-T cell expansion was accompanied by increase cleaved caspase-3 expression and a decreased ratio of EGFRvIII/EGFR in GBOs, thus suggesting that EGFRvIII+ cells were targeted and killed by CAR-T cells.

### Studies on tumor evolution and heterogeneity

Tumors are highly heterogeneous, and substantial heterogeneity exists in cancers across patients (intertumor heterogeneity) and within single tumors (intratumor heterogeneity). Tumor heterogeneity is an ongoing challenge in cancer medicine. Through construction of many PDO lines, the molecular pathologies of various tumors can be analyzed.

Yan et al.^96^ have constructed a GC organoid biobank comprising normal, dysplastic, cancer, and lymph node metastases (*n* = 63) from 34 patients. The success rate of normal organoids was more than 90%, and that of GC organoids exceeded 50%. Although the tumor purity in tumor tissues was variable, the organoids had a tumor purity greater than 90% in 95% of cases. This GC organoid library comprised the following common GC subtypes: microsatellite instability, intestinal (chromosome instability, CIN), diffuse (genomically stable), and Epstein–Barr virus. The authors studied intratumoral heterogeneity by constructing organoids comprising adjacent dysplasia, different tumor regions, and lymph node metastases from the same patient. They found that in one patient, tumor organoids from the area of dysplasia at the tumor edge and invasive area shared common early driver mutations involving TP53 and APC, but evolved to have very different patterns of chromosomal aberrations and unique driver mutations. Dysplasic tissues had ASPM, BAX, and TSC1 mutations, whereas invasive tumors had STK11 and SMARCA4 mutations. Moreover, extensive CIN was observed in dysplastic tissues and differed substantially from that in invasive tissues. A comparison of lymph node metastasis organoids with primary tumor organoids revealed marked differences in the mixed-type GC: only 23.3% of mutations were commonly shared, including the key driver TP53. These results indicate the importance of CIN in driving intratumoral heterogeneity and tumor progression.

Sachs et al.^59^ have established more than 100 BC organoids comprising primary and metastatic BC from 155 patients, achieving a success rate greater than 80%. They have analyzed the histology and receptor status, genomic characterization, and gene expression of the organoid library. BC organoids maintained the histology, genomic, and transcriptomic characteristics of the primary tumors. For example, ductal carcinoma generally gave rise to solid, coherent organoids, whereas lobular carcinoma mainly generated noncohesive organoids. Control organoids established from histologically normal preventive mastectomy samples were very well organized and displayed only a mildly complex cribriform architecture. In contrast, malignant BC organoids exhibited typical cancerous features, such as enlarged and polymorphic nuclei, high mitotic activity, apoptosis, and vacuole formation. In these cases, tumors positive for ER and/or PR generated 75% ER- and/or PR-positive BC organoids. ER- and/or PR-negative tumors generated > 90% ER- and/or PR-negative BC organoids. Eighty percent of HER2-positive BC tissue-formed organoids were HER2 positive, and 90% of HER2-negative BC tissue-formed organoids were HER2 negative.

Jacob et al.^89^ have generated a GBO biobank derived from 53 patients (including subregional samples) carrying a variety of genomic alterations commonly found in glioblastomas. The organoids were subjected to H&E staining, immunohistochemical staining, and RNA sequencing, thus revealing that the GBOs maintained not only their heterogeneity but also the molecular signatures of primary tumor tissues. For example, quantitative analysis of 8 tumor samples showed that in most cases, the percentages of cells expressing Sox2 and Olig2 were similar in their parental tumors and corresponding GBOs, lasting for up to 4 weeks. The authors generated organoids from different subregions (ANT and PMS) of the same patient, and genomic analyses revealed subregion-specific genomic variants, such as a PTEN missense mutation and copy number loss of 6q and 16q in UP-7788-PMS GBO but not in UP-7788-ANT GBO.

Calandrini et al.^85^ have generated tumor and matched normal kidney organoids from more than 50 children with KCs of different subtypes, including Wilms tumors, malignant rhabdoid tumors, renal cell carcinomas, and congenital mesoblastic nephroma, with a success rate greater than 75%. The organoids maintained the phenotypic characteristics of the original tumor tissues, and different cell populations were sufficiently distinguished. For example, organoid culture 88T demonstrated distinct clustering of 3 populations. Two of these clusters reflected epithelial subpopulations and represented one stromal cell. In 51T organoids, one population represented epithelial cells, a second population reflected stromal cells, and the third was more complex, expressing markers of epithelial cells, stromal cells, neurogenesis, and nephrogenesis. In addition to retaining the cell populations, KC cancer organoids also recapitulated the diverse genomic landscape and epigenetic profiles of the original tumor tissue, such as copy number alterations, cancer gene mutations, mutational signatures, gene expression patterns, and DNA methylation patterns.

Histological and immunofluorescence analyses of human bladder cancer organoids showed substantial variations among organoids from different tumors^91^. For example, organoids from different patients were either solid or lumen containing. Different organoids had different morphological structures; for example, some organoids showed smooth circular or slender structures, whereas others exhibited very irregular morphologies.

## Future prospects and challenges

Herein, we have attempted to summarize the basic status and applications of the PDO biobanks that have been successfully constructed. Tumor cells are acquired through a wide range of sources, such as tissues retrieved through fine needle biopsy, ultrasound, computed tomography guidance, endoscopic biopsy, rapid autopsy, and surgical resection, and even circulating tumor cells. In fact, cancer hospitals can obtain these tissues very easily. Cancer is a highly heterogeneous disease, and the tumor characteristics of each patient, and even the characteristics of the same tumor tissue, are not exactly the same; thus, the collection of cancer subtypes from many individual patients and different tumor regions may enable tumor heterogeneity to be further studied. In addition, PDO biobanks provide sufficient resources to conduct large-scale high-throughput screening of cancer treatment drugs and provide more accurate medication guidance for cancer treatments. In terms of individual precision medical treatment strategies, the combination of genetic testing and organoids could improve the effects of medications and prevent patients from experiencing the adverse effects of ineffective anticancer drugs.

Despite the advantages of PDO biobanks, many difficulties must be resolved. First, not all tissues can be used to construct organoids. According to previous reports, the features of primary tumors, including the clinical stage, treatment conditions before organoid generation, and pathological diagnosis, do not influence the success rate; however, culture condition modifications according to different tumor types and molecular characteristics can affect the success rate. Second, technology standardization is difficult. Consequently, organoid technology is not widely used for drug development or diagnostics, and similarly, different culturing techniques are used across laboratories. To address this challenge, Brandenberg et al.^97^ have launched an organoid array technology approach with the potential to fully automate organoid culture for high-throughput and high-content organoid-based screening. Third, although the DNA methylation diversity and transcriptome status in each tumor are stable, reactions to anticancer drugs can differ within the same tumor, thereby indicating that epigenetic changes greatly affect gene expression in cells, causing tumor heterogeneity and drug reaction variations. Better methods are therefore required for tracking and analyzing intratumoral heterogeneity. Fourth, organoids include only the epithelial layer and lack the surrounding mesenchymal cells, immune cells and nervous system^98^. A possible solution may be coculturing organoids with other cellular components, such as immune cells, stromal cells, and nerve cells^99^. Fifth, the construction and handling of living cancer biobanks is more tedious than that of traditional cancer biobanks, and essential growth factors are often expensive. To date, no standard protocols exist for organoid culture. However, as commercial entities begin to sell reagents and optimized methods suitable for organoid culture, standard protocols may become available. Sixth, the success rate of organoids is less than 100%; consequently, only some people benefit from personalized treatment with tumor organoids. Seventh, owing to the lack of relevant legislation, standards, and guidelines for organoid technology and tumor organoid banks in China, some problems associated with ethical approval and informed consent exist regarding the construction of PDO biobanks.

Although numerous questions remain, the construction of cancer patient-derived living biobanks is necessary for clinical and basic research. First, technological developments will gradually improve and standardize the construction methods. Second, an increasing number of tumor patients can provide tumor samples of different types and clinical stages. Third, big data analysis based on PDO living biobanks can enhance tumor prevention, diagnosis, treatment, and rehabilitation strategies.
